# Sepsis progression and outcome: a dynamical model

**DOI:** 10.1186/1742-4682-3-8

**Published:** 2006-02-15

**Authors:** Sergey M Zuev, Stephen F Kingsmore, Damian DG Gessler

**Affiliations:** 1 DFA Capital Ltd/AG, Norbertstr. 29, D-50670, Cologne, Germany; 2 National Center for Genome Resources, 2935 Rodeo Park Drive East, Santa Fe, NM 87505, USA

## Abstract

**Background:**

Sepsis (bloodstream infection) is the leading cause of death in non-surgical intensive care units. It is diagnosed in 750,000 US patients per annum, and has high mortality. Current understanding of sepsis is predominately observational and correlational, with only a partial and incomplete understanding of the physiological dynamics underlying the syndrome. There exists a need for dynamical models of sepsis progression, based upon basic physiologic principles, which could eventually guide hourly treatment decisions.

**Results:**

We present an initial mathematical model of sepsis, based on metabolic rate theory that links basic vascular and immunological dynamics. The model includes the rate of vascular circulation, a surrogate for the metabolic rate that is mechanistically associated with disease progression. We use the mass-specific rate of blood circulation (SRBC), a correlate of the body mass index, to build a differential equation model of circulation, infection, organ damage, and recovery. This introduces a vascular component into an infectious disease model that describes the interaction between a pathogen and the adaptive immune system.

**Conclusion:**

The model predicts that deviations from normal SRBC correlate with disease progression and adverse outcome. We compare the predictions with population mortality data from cardiovascular disease and cancer and show that deviations from normal SRBC correlate with higher mortality rates.

## Background

Sepsis is defined as occurring in patients who have evidence of local infection and two or more signs of systemic inflammatory response syndrome (SIRS, comprising perturbation of heart rate, respiratory rate, central temperature or peripheral leukocyte count)[1-3]. Despite intensive medical therapy, severe sepsis has a mortality rate of 25–50%, and sepsis is the tenth leading cause of death[4,5]. Sepsis is the leading cause of death in non-cardiac intensive care units, and third leading cause of infectious death. Ominously, incidence of sepsis is increasing by 9% per annum, and total healthcare cost currently exceeds $20 billion per annum[6-8].

Sepsis is a highly dynamic, acute illness. Common causes of sepsis mortality are refractory shock, respiratory failure, ARDS (Acute Respiratory Distress Syndrome), acute renal failure, or DIC (Disseminated Intravascular Coagulation). Rate of progression of sepsis to organ failure, septic shock and death in individuals is highly heterogeneous and largely independent of the specific underlying infectious disease process. For example, case fatality rates in patients with culture-negative sepsis are similar to those with positive cultures[9]. Mortality rates in sepsis are, however, critically dependent upon disease staging. Current differentiation of local infection, SIRS, sepsis, severe sepsis and septic shock relies exclusively on static clinical indices[1,2,10]. These include the Sepsis-related Organ Failure Assessment (SOFA) score, the Acute Physiology and Chronic Health Evaluation (APACHE II) score, the Pediatric Risk of Mortality (PRISM III, in children) score and blood lactate level[11-16]. These disease severity classification systems can prognostically stratify acutely ill patients and guide treatment intensity guidance. They are predicated upon the hypothesis that the severity of an acute disease, such as sepsis, can be measured by quantifying the degree of abnormality of multiple, basic physiologic principles[13]. In turn, these indices are based upon the long-established principle of bodily homeostasis, and are determined by measurement and multivariate analysis of the most deranged physiologic values during the initial 24 hours following presentation. Their validity has been established in numerous studies that have demonstrated linear relationships in cohorts between index value and hospital mortality. These indices have also proven valuable as surrogate end-points for the evaluation of efficacy in clinical trials of investigational new drugs[17,18]. Clinical indices such as APACHE II, however, were not designed to guide individual patient treatment decisions. Furthermore, these indices are, in general, not dynamical, and were not developed to reflect changes in physiologic data collected over time. In a highly dynamic illness, the use of indices at disease outset is insufficient to guide ongoing clinical management. Furthermore, sepsis is highly heterogeneous in terms of pathogen, source of infection, associated comorbidity, course and complications, making clinical assessment quite difficult.

The need for rapid, accurate identification of disease progression in sepsis increased dramatically with the availability of several, novel treatment regimens. While novel sepsis therapies are improving sepsis outcomes, they are creating new patient management and diagnostic challenges for physicians. For example, in 2001 the Food and Drug Administration approved activated protein C (APC) for treatment of patients with severe sepsis and APACHE II score of ≥ 25. In the pivotal trial of APC (PROWESS), 28-day mortality was decreased by 6% (ref. [17]). The greatest reduction in mortality (13%) and cost effectiveness was observed in the most seriously ill patients (those with APACHE II score ≥ 25)[17,18]. In contrast, APC exhibited modest survival benefit and cost-ineffectiveness in patients with sepsis and APACHE II score < 25. However, APC therapy is associated with a 1–2% incidence of major bleeding. For these reasons, widespread, appropriate use of APC in sepsis is most likely to occur following deployment of an objective, accurate, rapid, dynamical model of sepsis.

Another recent therapeutic development that has shown significant potential to reduce sepsis mortality is early aggressive therapy to optimize cardiac preload, afterload, and contractility (Early Goal Directed Therapy, EGDT)[19]. Patients randomized to EGDT receive more fluid, inotropic support, and blood transfusions during the first six hours than control patients administered standard therapy. During the subsequent 72 hours, patients receiving EGDT had a higher mean central venous O_2 _concentration, lower mean lactate concentration, lower mean base deficit, and higher mean pH. Mortality was reduced by 16% in the EGDT group. A dynamical model of sepsis that provides rapid, quantitative, objective determination of the stage of sepsis development and likelihood of progression is needed to guide selection of patients for EGDT.

Other sepsis treatments that may improve survival include intensive insulin therapy (to maintain tight euglycemia), physiologic corticosteroid replacement therapy, protocol-driven use of vasopressors and rapid administration of appropriate antibiotics[20-22]. Given heterogeneity in disease progression in sepsis patients, however, evaluation of the value of novel therapies is greatly assisted by evaluation of surrogate end-points. Efficacy with many of these treatments appears limited to certain sepsis patient subgroups. Furthermore, most of these emerging treatments require careful patient selection and monitoring to avoid adverse events. Patient selection for these novel therapies would be greatly advanced by the availability of dynamical, data-driven models of sepsis that incorporate surrogate markers.

In summary, given the highly dynamical nature of critical, acute illnesses such as sepsis, the existence of multiple, alternate complications, and the availability of many therapeutic and treatment intensity options, there exists a pressing need for dynamical models of disease. Such models, like their static, predecessor indices, should be based upon a fundamental, comprehensive but dynamical understanding of the derangement of physiologic processes in sepsis. Unlike conventional clinical indices, however, their development should be tailored specifically to guide treatment decisions in individual patients, and should be predicated on changes in values observed in serial observations. Also in contrast to conventional indices, such models will be designed for clinical relevancy with excellent predictive value for the immediate future (in the case of sepsis, for 6 – 12 hours), rather than long-range predictive value (such as 28-day mortality in the case of conventional clinical indices). Indeed, efforts are underway by several groups to create mathematical models of sepsis[23,24], and to evaluate their usefulness in the design of clinical trials of investigational new drugs[25].

Recent advances in multiplexed measurement technologies for biomolecules, biomarker development and modeling of gene or protein networks or pathways in disease states are starting to be integrated with clinical and physiologic measures in human health and disease[26]. Reductionist analyses – division of physiologic states or disease systems into component variables and "solving" of differential equations for each with empiric data – are starting to yield dynamical models with predictive or prognostic value, both generally[27] and specifically for sepsis[23,24]. Although many biological systems are complex and non-linear, much of current biological knowledge was derived from deterministic, reductionist analyses[25,28]. Despite the underlying complexity of disease mechanisms, disease states are frequently associated with linear dynamics[29] (or, more accurately, with the breakdown of multi-scale fractal complexity). Reductionist methods are likely to remain useful for the foreseeable future for quantitative prediction of responses to perturbation of networks.

The current study represents a first step in the application of reductionist analyses to a dynamical model of the progression of sepsis in individual patients. The goal of such studies is to move from static, prognostic indices useful in sepsis cohorts to relatively simple, dynamical models that are useful in real-time guidance of treatment and treatment intensity at the bedside in individual patients with sepsis. An innovative, hybrid, infectious and vascular model of sepsis is presented that builds upon previous scaling models of vascular circulation[30-33] and includes variables such as age, end-organ damage, disease progression, and mortality.

We ground the modeling approach on fundamental processes of energy production and consumption. In a living body these processes comprise the energy metabolism made classic 40 years ago by M. Kleiber in his book "The Fire of Life"[34]. From the molecular and cellular point of view, the process of life is the process of interactions among particles – molecules of cytokines, glucose, oxygen, and others, among different cells, viruses, bacteria, and so forth.

A necessary condition for particle interaction is their contact. Two particles – a viral particle and an antibody, for example – must contact each other in order to interact. This contact or collision is possible due to their motion within the blood, lymph, or interstitial spaces, as the blood and lymph transport particles to interaction zones. An increase in energy production increases the oxygen consumption that is associated with a rise in the rate of blood and lymph circulation. This rate is a crude index of the intensity of biological life; it scales across taxa and with biological time, such as in the average life span and number of heart beats per life[30-33].

The above consideration leads to the recognition that the rate of blood circulation should play an essential role in disease origin and progression. For example, blood and lymphatic circulatory systems play important roles in the life of T-lymphocytes, as they migrate from the bone marrow, mature in the thymus, and act as effectors throughout the body. A similar dependency on circulation takes place during viral infections when infected cells produce new viral particles. Production of virions is restrained by destruction of infected cells by immune mechanisms, viral particle inactivation through humoral mediators, including antibodies, the complement cascade and cytokine elaboration, and decreased viral replication through humoral mediators or therapeutic agents. A prerequisite of these responses is physical interactions between cells, viral particles and blood proteins. While a high rate of fluid circulation enhances such interactions, it also enhances viral and immune effector dissemination. This can lead to organ damage both through viral cytopathology and through inflammation. Thus low or high circulation rates may both be sub-optimal in relation to the competing demands during sepsis progression. A pioneering example of cellular and humoral factor interaction models to explain the dynamics of sepsis progression used agent-based modeling[35,36], rather than the reductionist approach, described herein.

In the present paper, we have formalized this relationship between circulatory and interaction events based on the earlier work of ref. [37]. The parameters of the model present the intensities of interactions among immune and infectious components by incorporating the rate of blood circulation as mentioned above. Thus the basic assumptions rely on the well known modeling techniques of particle interaction under systemic and Brownian motion (see below).

## Results

### Rate of blood circulation and body size

We consider the well established correlation between the rate of blood circulation and body mass[38]. In general, the following allometric relation is widely supported across taxa[32,39,40]:

*V *= *q*·*m*^3/4 ^    (1.1)

For humans, the coefficient *q *is approximately 0.256 [ref. [38]]. *V *and *m *are rate of blood circulation (liter/min) and body mass (kg), respectively[32]. It should be noted that equality (1.1) applies to individuals that have little or no "redundant" body mass – that which has no clearly attributable physiologic function, or the continuum of mass in excess of ideal body weight, obesity and morbid obesity. Redundant body mass is not necessary for normal functioning of the individual, but increases the volume of the circulatory system, thereby increasing demands on cardiac output.

We incorporate redundant body mass explicitly with the following supposition:

**Supposition 1.1. **The human body mass *M *may be presented as the sum:

*M *= *m *+ *R*,     (1.2)

where *m *and *R *are the basal (or ideal) and redundant body mass, respectively, and equality (1.1) is true for the basal body mass *m*. Ignoring the effect of sex and size of frame, the basal body mass is the mass that provides a normal living activity of a body of height *h*(cm) and is defined as[41]:



Resting on this supposition we can rewrite (1.1) as



Then, for a mass-specific rate of blood circulation *v *we have:



If redundant body mass *R *is equal to zero then *M *= *m*, and



According to (1.5) the mass-specific rate of blood circulation (SRBC) depends on two parameters: *h *and *M*. It is convenient to express the influence of redundant body mass on the SRBC with the ratio:



which presents the relative SRBC with respect to an ideal body of the same height.

**Definition 1.1. **For any given patient, one can construct a reference or *basal individual*, *i.e*. an individual having the same height and *Q *= 1. In this patient relations such as (1.1) and (1.3) are valid, in agreement with the underlying model[32]. It follows from (1.5) and (1.6) that



Using the new variable



which presents the percent of redundant body mass in the patient under consideration, we obtain:

*Q *= (1 + *r*/100)^-1^.     (1.8)

**Figure 1:**
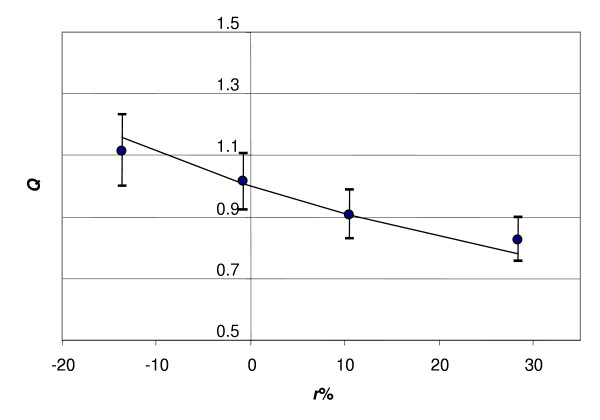
**Correlation between *Q *and redundant body mass (*r*%)**. Solid line is equation (1.8); dots are average values of *Q *(1.9) with a least squares fit to (1.8) yielding *q *= 0.233. Each point presents the average value calculated from 15 observations. Error bars represent 95% confidence intervals.

We verify (1.8) by considering the correlation between *Q*, calculated from rate of blood circulation according to (1.7):



and the percent of redundant body mass calculated as



where *m *is given by (1.3). The value of *q *in formula (1.9) is calculated using a least squares fitting of the theoretical dependence (1.8) and the observed correlation between *Q *(1.9) and *r*% (Fig. 1). The data for the figures in this manuscript are from volunteers enrolled by the Moscow State Medical Academy (Russia, courtesy of Dr. V. K. Korneenkov). Body mass (kg), height (cm), lung capacity (L), fasting glucose concentration (mmol/L), rate of blood circulation (by echocardiography, in L/min), and cardiac stroke volume (by echocardiography, L) were measured in 82 healthy males and females, aged 17 – 65 years.

**Figure 2:**
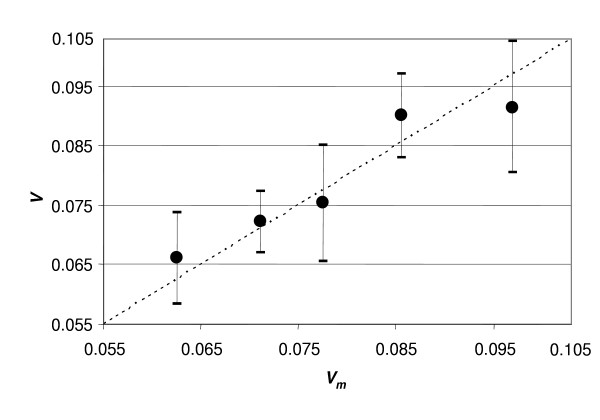
**Correlation between observed SRBC (*v*) and its estimate (*v*_*m*_) calculated from height**. The estimate *v*_*m *_is calculated from equ. (1.10). Each point presents the average from 12 cases. Dashed line presents *v *= *v*_*m*_.

The agreement of equation (1.8), derived from (1.5) and (1.6), with the data supports equation (1.5) and ultimately Supposition (1.1). However, there is also direct evidence for the validity of Supposition (1.1). Consider the two variables:



If Supposition (1.1) is true the correlation between these variables must be linear (Fig. 2).

Thus the rate of blood circulation is strongly correlated with body height and, because this is mass-specific, it does not change appreciably as body mass decreases or increases. Thus we expect redundant body mass to present a detrimental load relative to the individual's height. We take this feature into account using specific rate of blood circulation (1.5).

We note that *Q *(1.7) is inversely proportional to body mass index (BMI)[42]. This follows immediately from (1.3), (1.5) and (1.6) if we take into account that BMI uses the measurement of height in meters:



The BMI is widely used in studies of human health. It is known, that the values of BMI between 20 and 25 are generally correlated with a healthy state. Either increased or decreased BMI with respect to a reference group (persons with *BMI *of 22–23.9) corresponded to a rise in the risk of death from all causes, though the increase needed to be substantial (*BMI *≥ 32; an increased BMI from 23.9 to 32 did not show a significant increase in risk)[43]. It follows from (1.11) that the same conclusion should be applicable to *Q*. In turn, according to the definition of *Q *(1.7), this is associated with the variation in mass specific rate of blood circulation, *i.e. *this risk is minimal when *v *= *v*.

### Rate of blood circulation and particle interaction

The previous conclusion allows the inference that rate of blood circulation plays an essential role in disease origin and progression. In order to study this phenomenon let us consider how the rate of blood circulation influences the intensity of molecular interactions in blood or interstitial fluid.

We consider an intercellular space (zone of interaction) in a patient with sepsis (bloodstream infection), where particles (viral particles, molecules of antibodies, cytokines, complement and coagulation factors, and others) move and interact within the surrounding fluid. In order to create the model let us describe the trajectory of a particle along the direction of fluid motion in this zone.

Since intercellular space is considered an inhomogeneous environment, we distinguish two components of particle motion – its drift and diffusion. The first component presents the systematic pressure on the particle travelling together with the fluid flow; the second describes the particle's random motion within this flow. We can suppose that due to the inhomogeneous structure of the intercellular space, the particle's motion among unmoved cells, and collision with other particles inside the flow, constitute properties of Brownian motion. According to this, for the increment of the particle's coordinate during small interval Δ*t *we write:

*z*(Δ*t*) = *a*(*v*)Δ*t *+ *b*(*v*)·*w*(Δ*t*),     (2.1)

where first term in the right-hand site describes the drift, second one presents diffusion, and *w*(*t*) is the Wiener process[44].

It is natural to equate the systematic pressure of the drift term as proportional to SRBC, and thus we can write:

*a*(*v*) = *a*_0_·*v*,

where *a*_0 _> 0 is a constant. In order to obtain *b*(*v*) recall that the coefficient of diffusion, *d*(*v*) = *b*^2 ^(*v*), is proportional to the kinetic energy of the particle, *i.e*.,

*d*(*v*) = *b*^2 ^(*v*) = ·*v*^2^,

where *b*_0 _= 0 is a constant. Therefore, we can rewrite (2.1) as

*z*(Δ*t*) = *a*_0_·*v*·Δ*t *+ *b*_0_·*v*·*w*(Δ*t*).     (2.2a)

Consequently for the basal patient we have:

*z*(Δ*t*) = *a*_0_·*v*·Δ*t *+ *b*_0_·*v*·*w*(Δ*t*),     (2.2b)

where underlining indicates the basal patient.

Using the parameter



we can rewrite equations (2.2) in the following form:

*z*(Δ*t*) = *a*(*v*)··Δ*t *+ *b*(*v*)··*w*(Δ*t*),     (2.4a)

and

*z*(Δ*t*) = *a*(*v*)·Δ*t *+ *b*(*v*)·*w*(Δ*t*),     (2.4b)

where *a*(*v*) = *a*_0_·*v* and *b*(*v*) = *b*_0_·*v* characterize the drift and diffusion in the basal patient. Therefore, for the drift and diffusion coefficients we have

*a*(*v*) = ·*a*(*v*) and *d*(*v*) = *H*·*d*(*v*).     (2.5)

Equations (2.4) and (2.5) are the starting relations where the following results are proved[45].

*Lemma 2.1. *For both the system studied and the basal system the increments in the coordinates satisfy the equalities:

*u*(Δ*t*) ≐ *u*(Δ*t*·*H*), *u*(Δ*t*) ≐ ·*u*(Δ*t*),

where symbol ≐ means stochastic equivalence, and

*u*(Δ*t*) = *z*(Δ*t*) - *a*(*v*)··Δ*t *= *b*(*v*)··*w*(Δ*t*),

*u*(Δ*t*) = *z*(Δ*t*) - *a*(*v*)·Δ*t *= *b*(*v*)·*w*(Δ*t*)

describes the particle motion within the fluid flow in the studied and basal patients.

The particle contacts which lead to their interactions result from their diffusion motion. The intensity of particle interactions λ is defined as the average number of interactions per unit of time:



where E is the mathematical expectation and *n*(Δ*t*) is a random number of the particle interactions in Δt.

Using *Lemma 2.1 *we prove the following statement:

*Lemma 2.2. *The intensities of interactions in the system studied and the basal system satisfy the relation:

λ = *H*·λ.

Let *x*_*t *_be the concentration of particles of some kind in zone of interaction at time *t*, and *X*_*t*_, be their number. By the definition

*X*_*t *_= *U*·*x*_*t*_,

where *U *is the effective volume of interactions, *i.e.*, the measure of the domain Ω, which is formed in the fluid flow by moving particles. In this case the following proposition may be proved:

*Lemma 2.3. *The effective volumes of interaction *U*, *U* in the system studied and in the basal system respectively satisfy the condition:



*Lemma 2.4. *The stationary concentrations *x*_∞_, *x*_∞ _and the number of particles of some kind *X*_∞_, *X*_∞ _in the system studied and in the basal system are related by:

*x*_∞ _= *H*^-1/2^*x*_∞_,

*X*_∞ _= *H*·*X*_∞_..

Let us suppose now that the state of a system of interacting particles at time *t *is characterized by the vector *x*_*t *_∈ *R*^*N*^, whose components are concentrations of interacting particles of *N *kinds. We assume that the stationary state *x*_∞ _is steady and the response of the system to an external disturbance *g *in time *T *is described by the system of ordinary differential equations:



where *f*(•,•,•) is a continuous vector-function that describes the entry of particles, the structure of their interactions, and the utilization of complexes; α ∈ *R*^*L *^is the vector of positive parameters. This vector takes into account the interactions between particles with components that are proportional to the intensity of interactions λ, defined as the limit (2.6).

*Theorem 2.1. *If the relationships obtained in lemmas 2.1 – 2.4 are valid, the change in the state of the system studied is described by a model in the form (2.7) which contains only the base parameters and *H*:



where

 or taking into account (1.7) *H *= *Q*^2^.

Theorem 2.1 allows us to study how the mass-specific rate of blood circulation influences disease progression (Section 5). First, however, let us consider the correspondence of these results to the data and find out how the value of *H *may be estimated from physiological indices.

### Estimation of *H *from physiological measurements

The first formula for *H *follows directly from Lemma 2.4. Indeed, let *g *and *g*be the concentrations of fasting glucose in the studied patient and in the basal patient respectively. According to Lemma 2.4 we have:



where *g*is the homeostatic concentration[46] (from 3.3 to 6.1 mmol/L).

**Figure 3:**
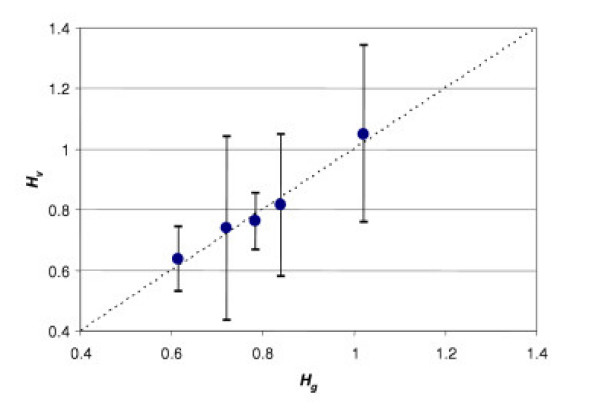
Correlation between two estimates of *H*: *H*_*g *_*vs. H*_*v*_. *H*_*g *_is calculated from fasting glucose concentration; *H*_*v *_is calculated from the specific rate of blood circulation. Each point presents the average value calculated from eight observations for *q *= 0.256 and *g*= 4.05 mmol/L.

To test this, consider the definition  and the two estimates  and . Since these two variables both estimate *H*, the correlation between them must be linear; moreover, it must correspond to the relation *y *= *x *(Fig. 3).

In order to test Lemma 2.3, let us suppose that effective volume within which molecules of oxygen interact with erythrocytes is proportional to lung capacity *W*. It follows from Lemma 2.3 that



where *W*= 0.058·*h *- 4.788 for males and *W*= 0.038·*h *- 2.468 for females [47].

**Figure 4:**
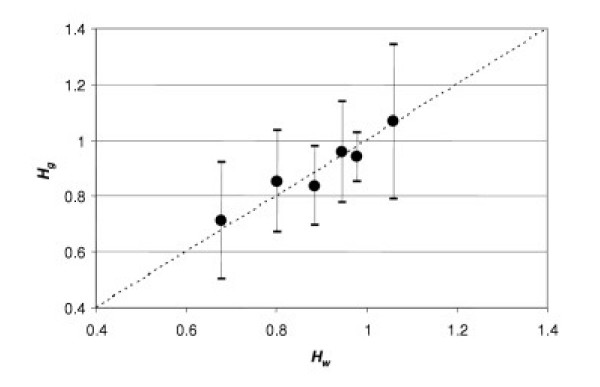
**Correlation between two estimates of *H*: *H*_*g *_*vs. H*_*w*_**. *H*_*g *_is calculated from fasting glucose concentration; *H*_*w *_is calculated from lung capacity. Each point presents the average value calculated from seven observations for *g*= 3.9 mmol/L.

If our supposition is true we will obtain a linear dependency between *H*_*g *_and *H*_*w *_(Fig. 4).

One more formula gives us the result obtained in Section 1. Since according to (1.8)



and



**Figure 5:**
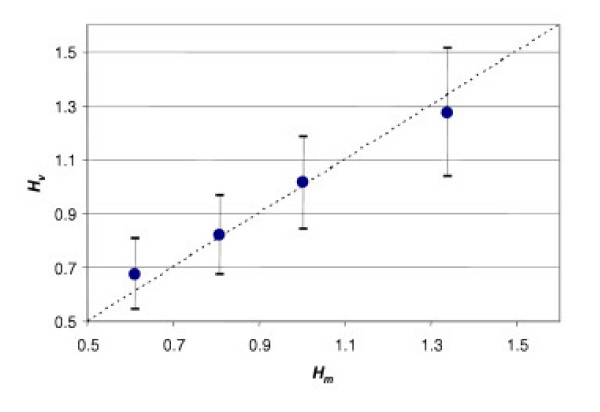
**Correlation between two estimates of *H*: *H*_*v *_*vs. H*_*m*_**. *H_v_*is calculated from the specific rate of blood circulation;   *H_m_* is calculated from body mass. Each point presents the average value   calculated from 15 observations for *q* = 0.236.



Fig. 5 presents the correlation between *H*_*v *_and

Thus from (1.11) and (3.3) we have . As we noted in the end of Section 1, either increased or decreased BMI with respect to a reference group (persons with *BMI *of 22–23.9) corresponds to a rise in the risk of death from all causes. Therefore, as *H *deviates from unity, it indicates an increased risk of disease origin.

### Application to disease modeling in sepsis

To apply the results obtained in Section 2 we use our modification of the "Simple Model of an Infectious Disease" that takes into account the main principles of disease dynamics[37]. This model consists of four differential equations:



where *P*(*t*) is the concentration of a pathogen at time *t *(*t *= 0 is the moment of infection), *F*(*t*) is the concentration of "humoral factors" – a summarized effect of innate and cognate immune defense (cytokines, interferons, complement and coagulation cascades, pentraxins, antibodies, etc.), *C*(*t*) is the concentration of various cells that elaborate humoral factors (especially leukocytes, platelets and endothelial cells), and *D*(*t*) is a relative characteristic of an organ's damage, 0 ≤ *D*(*t*) ≤ 1. The values *D*(*t*) = 0 and *D*(*t*) = 1 correspond to the healthy state and complete organ failure respectively. The negative influence of the damage on the ability of the patient to resist an infection is taken into account by function ξ(*D*) (third equation of system [4.1]). If 0 ≤ *D*(*t*) ≤ 0.1 then ξ(*D*) = 1, if 0.1 <*D*(*t*) ≤ 0.75 then ξ(*D*) = exp{-7.5(*D *- 0.1)}, and if *D*(*t*) > 0.75 then ξ(*D*) = 0, *i.e*., we consider that the patient is unable to resist when 75% or more of organ function is ablated. Table 1 summarizes the model's parameters[34].

**Table 1:** Parameters for Circulation, Infection, Recovery Model Parameters used in systems (4.1) and (4.2).

Parameter	Interpretation
β	Pathogen rate of reproduction
σ	Pathogen virulence and cytotoxic action of T-lymphocytes
γ	Intensity of a pathogen binding
ρ	Intensity of antibody production
α	Intensity of plasma cell production
η	Number of antibodies needed to neutralize a single antigen
	Average antibody lifespan
	Average plasma cell lifespan
μ_*m*_	Host recovery rate
τ	Period of time needed for the clone formation
*C*^∞^	Homeostatic concentration of plasma cells
*P*^0^	Initial concentration of a pathogen

Model (4.1) differs from the previous model [37] by the first term in first equation. In the original model this term is β·*P*, which does not model the rate of pathogen reproduction as being proportional to the undamaged part of the organ's function. In the model of (4.1) an increase in damage suppresses pathogen reproduction. We also use a modified fourth equation, with σ·*P*·*F *instead of σ·*P *because *F*(*t*) presents a summarized effect of immune defense, including immunopathology that further impairs organ function (e.g. T lymphocyte-mediated immune destruction of an organ's cells).

Let us apply now Theorem 2.1 to this model in order to study how SRBC influences disease progression. Applying formula (2.8) to system (4.1) we have:



where *H *> 0 takes into account individual features of the patient under consideration, and parameters {γ, ρ, μ_*F*_, μ_*C*_, μ_*m*_, α, τ, *C*^∞^, *F*^∞^} correspond to the basal patient.

It may be noted that for the delayed variable , we now have  by applying equation (2.8) to the system that describes the effect of delay as shown in ref. [37].

We note that for computational experiments it is more convenient to use dimensionless variables:

*X*_1_(*t*) = *P*(*t*)/*P*(0), *X*_2_(*t*) = *F*(*t*)/F*, *X*_3_(*t*) = *C*(*t*)/C*, *X*_4_(*t*) = *D*(*t*).

For these variables we have from (4.2):



where the parameters *a*_1_, *a*_2_, ..., *a*_8 _correspond to the basal patient.

In order to study the influence of SRBC on disease progression and its outcome, let us consider the case where the values of the basal patient's parameters provide a solution to system (4.3) that is interpreted as a sub-clinical form of a disease. For the basal patient we set *H *= 1, with constant parameters[48]:

*a*_1 _= 19.2, *a*_2 _= 22.1, *a*_3 _= 0.17, *a*_4 _= 8.0·10^-6^, *a*_5 _= 0.1, *a*_6 _= 0.5, *a*_7 _= 9.2·10^-3^, *a*_8 _= 0.12, τ = 0.5.

We then analyze the quantitative change of the solution versus *H*.

**Figure 6:**
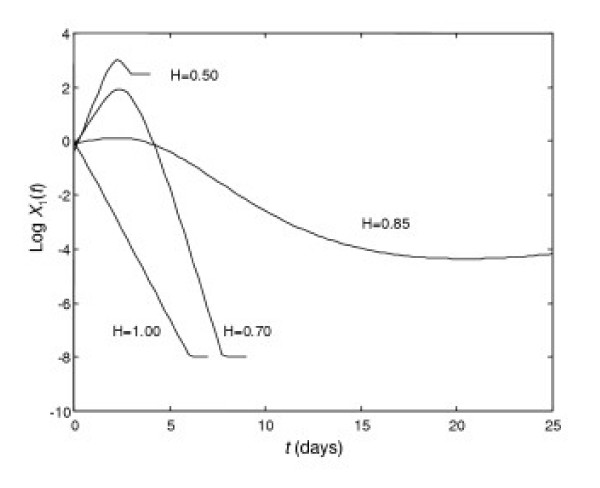
**Dynamics of the relative concentration of the pathogen at different values of *H***. *H *= 1 – sub clinical form, *H *= 0.85 – chronic form, *H *= 0.7 – acute form, *H *= 0.5 – lethal outcome. Y-axis is the log(*X*_1_(*t*)).

The results are presented in Fig. 6 for the variable *X*_1_(*t*) = *P*(*t*)/*P*(0) – the relative concentration of a pathogen. Accordingly, *H *= 1 corresponds to sub-clinical disease, while a decrease in *H *results in an indolent or chronic form of disease (*H *= 0.85). A further decrease in *H *leads to an acute form of disease (*H *= 0.7). As *H *decreases considerably (*H *= 0.5) we obtain a lethal outcome because end-organ damage *X*_4_(*t*) = *D*(*t*) has reached the upper bound *D*(*t*) = 0.75 that corresponds to 75% impaired function (data not shown).

Fig. 6 also shows that we stopped our calculations when relative concentration of the pathogen *X*_1_(*t*) reached the value 10^-8^, *i.e*., when *P*(*t*) ≤ *P*(0)·10^-8^. The horizontal parts of the lines indicate a halting of the calculations. Thus, a decrease in *H *leads to disease development, and even to mortality. It should be noted that in the case considered, a further increase in *H* (*H *> 1) increases the rate of the pathogen elimination, *i.e*., the negative slope of the *H *= 1 line in Fig. 6. In some cases though, it may lead to a lethal outcome for a patient with different immune system parameters. Indeed, let us consider the case where *a*_2_, a measure of the affinity of host antibodies to the pathogen, is decreased, but where *a*_5_, the rate of plasma cell production (antibody producing cells), is increased. In order to simulate this case, the following parameters are instructive:

*a*_1 _= 0.50, *a*_2 _= 0.14, *a*_3 _= 0.17, *a*_4 _= 8.0·10^-6^, *a*_5 _= 5.5, *a*_6 _= 0.5, *a*_7 _= 9.2·10^-3^, *a*_8 _= 0.12, τ = 0.5.

**Figure 7:**
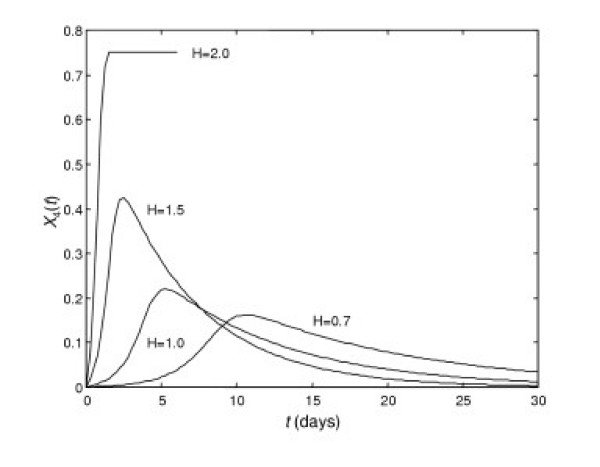
**Dynamics of organ damage during a disease at different values of *H***. Increase in *H *leads from acute disease forms to lethal outcome. Y-axis is *X*_4_(*t*).

Here we simulate a stronger immune response as the rate of plasma-cell production (*a*_5_) is increased from 0.1 to 5.5. At the same time, the affinity of free pathogen binding (*a*_2_) is diminished from 22.1 to 0.14. Thus, this example could represent more abundant antibody production, but of lower affinity. In this case, even for a pathogen having a lower rate of multiplication *a*_1_, we can obtain a lethal outcome by raising the value of *H *as shown in Fig. 7.

Fig. 7 shows that in the case when patients produce more antibodies, but of lower affinity, patients having a low mass-specific rate of blood circulation (low values of *H*) incur less intense organ damage because a low rate does not provide, for example, pathogen spreading to or within organs (such as lung parenchyma in community-acquired pneumonia, or CAP).

Therefore, either an increase or decrease in *H *can lead to a lethal outcome (see Section 2 taking into account *H *= *Q*^2^). This fact is used in the mortality model [47] that describes the age specific mortality rate in a population.

### Application to mortality modelling

**Figure 8:**
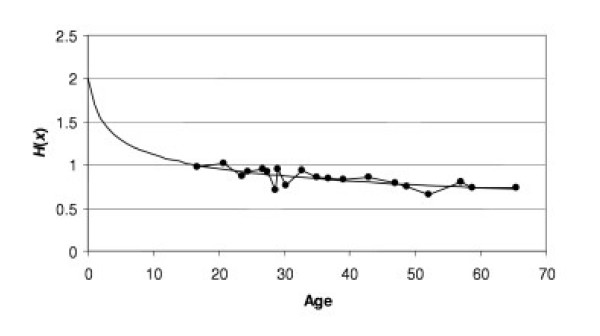
**Typical age specific dynamic of *H***. Each dot presents the average from 4 cases; solid line corresponds to equ. (5.2) when *a *= 2.0021, *b *= 0.2428, *H*_0 _= 0.8953, λ = 0.0026, *X*_0 _= 65.5076.

In this section we use a mortality model [47] with an aim to interpret *H *with respect to age. The mortality rate as the function of age *x *may be presented in the following form [47]:

μ(*x*) = μ_0_·[exp{16·(*H*(*x*) - 1)} + exp{-16·(*H*(*x*) - 1)}] + *c *    (5.1)

with a minimum at *H*(*x*) = 1. The dependence of *H *on age is presented by the monotonically decreasing function:



where *a*, *b*, *H*_0_, λ, *X*_0 _are parameters (Fig. 8).

From equation (5.2) we can calculate the age specific mortality rate (5.1) for this small population under the supposition that the minimal mortality rate 2μ_0 _and background mortality *c *in this sub-population are the same as in the population in a whole.

Conversely, having mortality model (5.1) we can calculate curve *H*(*x*) for the population using the mortality rate in the population. Let us analyze this curve given that



in accordance with the results of Section 3. Of course, we can suppose that *H*(*x*) is calculated from lung capacity or glucose concentration but we have chosen body mass because this parameter is more convenient for this interpretation.

**Figure 9:**
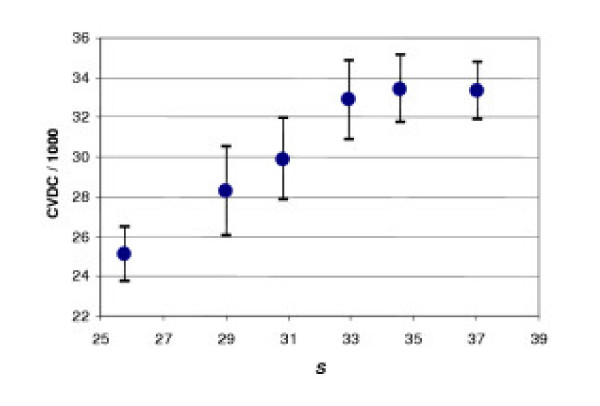
**Correlation between the total mortality from cardiovascular diseases plus cancer (CVDC; annual number of cases) and the measure of discrepancy (*S*). ***S *(equ. 5.3) is calculated from female and male mortality rates in Sweden (1951 – 1992). Each point presents the result of averaging (14 cases) for close values of *S*.

As shown in Fig. 8, during youth *H*(*x*) > 1 and consequently  because the growth of body mass lags behind increases in height. In an ideal case, when height stops increasing, for example at age *x *= a*, body mass will reach the value  and also stop increasing. In this ideal case we would have the following dynamics for *H*: as *H*(*x*) decreases in accordance with (5.2) and reaches the value *H*(*x*) = 1 at the age a*, *i.e*., *H*(a*) = 1, and then it remains at this level, *i.e.*, *H*(*x*) = 1 for all *x *> a*. However, Fig. 8 shows that *H*(*x*) continues to decrease after a*. Therefore, the difference 1 - *H*(*x*) for *x *≥ a* may be used as a measure of discrepancy between the ideal and observed curves. More exactly, we use the following integral that summarizes all differences:



Fig. 9 shows the correlation between this measure and total mortality from cardiovascular diseases and cancer (CVDC) in Sweden (In [5.2] *X *= 90 years). In order to plot this diagram we used mortality rate curves (females and males) for each year from 1951 to 1992 that gave us 84 pairs (*S*, *CVDC*). This diagram indicates that mortality from CVDC increases with discrepancy *S*.

In turn, the area *S *increases as age a* diminishes. In contemporary Sweden, for example, a* < 10. However, it is clear that at this age body height continues to increase, and consequently the inequality *H*(*x*) < 1 means that after age a* body mass exceeds that predicted from height alone.

**Figure 10:**
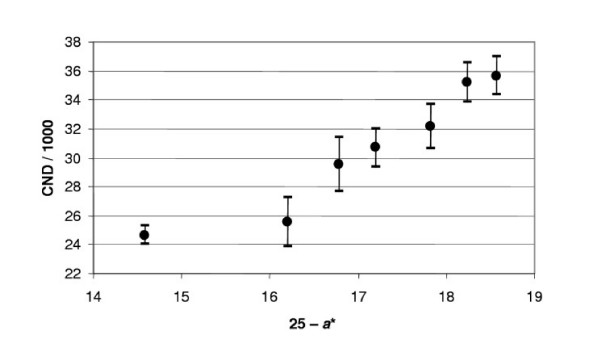
**Correlation between the total mortality from cardiac and neoplastic diseases (CND; annual number of cases) and 25 less the age when adult height is first attained**. The difference (25 - a*), where a* is the age when height stops increasing, is calculated from female and male mortality rates in Sweden (1951 – 1992). Each point presents the average from 12 observations.

If we suppose that the process of growth is substantially completed by the age 25, then we can expect that around this age physiological parameters will be adjusted with each other including height and body mass. Therefore, in the neighborhood of this age the value of *H *must be close to 1. Since a* < 25 let us consider the difference 25 - a* as independent variable instead of *S *in our previous data analysis (Fig. 10). As the above mentioned discrepancy appears at the age a* < 25 and then increases with age, Fig. 10 indicates that the earlier it appears, the higher is the total mortality from cardiovascular diseases and cancer in the population. This conclusion is related not only to body mass but is also related to the mass-specific rate of blood circulation, fasting glucose concentration, and lung capacity – the physiological indices that, according to Section 4, allow us to assess the correspondence of the studied patient to the base patient using *H*.

## Discussion

Recent advances in bioinformatics and in the precision of technologies for comprehensive measurement of biomolecules have created the novel discipline of systems biology[26]. Defined as the analysis all of the biomolecules in an experimental system simultaneously, systems biology is creating the opportunity, for the first time, to understand the molecular cause and course of complex traits, including sepsis[49]. Clinical systems biology, or the application of systems biology to human disease, holds considerable promise for personalized medicine. One of the first dividends of clinical systems biology has been the identification of novel biomarker candidates – biological markers that correlate with disease diagnosis, prognosis, staging, progression or drug efficacy. The promise of clinical systems biology is the validation of a subset of biomarkers as surrogate end-points and their use in guiding individual patient management. The key to deployment of clinically useful surrogates is their incorporation into robust, dynamical models of disease that enable real-time, data-driven, treatment decisions. Diseases and clinical states which are excellent candidates for initial implementation of dynamic, data-driven patient management are those where intensive physiologic monitoring and treatment algorithms are already established. They include labor and delivery, acute myocardial infarction, cardiac arrest, and sepsis[50].

Concurrent with the maturation of clinical systems biology, mathematical models are increasingly contributing an integral role to a dynamic understanding of human anatomy[51], physiology[54], and pathology[53-56]. Hitherto, however, few examples exist of hybrid models (that incorporate contributions by more than one organ system; though see, for example, ref. [35]). The present article describes a dynamical model that represents a basic theoretical framework for understanding how individual progression in a systemic disease, such as sepsis, can be driven by variations in both the immune and circulatory systems.

Critical to the development of clinically useful, dynamical models of sepsis is the recognition of strong and consistent underlying relationships between acute physiologic derangement and the risk of death during sepsis[13,57-61]. In particular, the concept of mass-specific rate of blood circulation has been introduced because the rate of blood circulation is strongly correlated with body height while being relatively independent of changes in patient body mass (Section 1). Consequently, a mass-specific rate of blood circulation decreases with the body mass growth. The metric, *H*, was proposed, that is the square of the ratio of the actual to the ideal mass-specific rate of blood circulation. Deviations of *H *from unity lead to unfavorable disease progression and outcomes since these deviations influence the intensity of particulate movement and interaction in the blood and interstitial fluid (Section 2).

The parameter *H *could be considered as a quantitative measure of the correspondence of a patient to an ideal or basal individual and therefore, it may be used to establish basal expectations for health monitoring with the aim of disease prevention. To this end, estimates of *H *calculated from easily measured physiological characteristics such as body mass, height, fasting glucose concentration, and lung capacity may be used (Section 4). As *H *deviates from unity, it indicates an increased risk of disease origin, unfavorable course and outcome. Understanding the basic expectations of disease progression as affected by deviations in blood circulation would start to allow partition the variance of observed cases (*i.e*., normalizing responses for differences in height and weight), thus increasing the predictive power of biomarker-based models.

Predictions from this model were compared with population mortality data from cardiovascular disease and cancer (Section 5) and showed that, upon adjustment for age, deviations from normal SRBC correlate with higher mortality rates. Current, prospective studies will provide an additional cohort in which to evaluate predictions from this model.

In accord with this model, clinical studies and recent therapeutic advances strongly suggest that vascular or microcirculatory perturbations represent a major determinant of mortality in sepsis[50,65-68]. Septic shock (the failure of circulatory homeostasis due to sepsis) is associated with 50% mortality[7]. Major improvements in mortality in severe sepsis have been achieved by algorithm-based use of vasopressors, early goal directed therapy, and infusion of activated protein C. Each of these interventions has a major mechanism of action that is based upon restoration of adequate blood microcirculation[62,63].

Another advance in the present model is the introduction of end-organ insufficiency and failure, rather than just mortality (Section 5). The clinical course in sepsis is highly variable and insufficiency or failure in a variety of organs can contribute to morbidity or mortality. For example, insufficiency in any of five end-organs is sufficient to meet the definition of severe sepsis, which is associated with a substantial increase in mortality.

Another feature of the present model that will improve clinical utility in prediction of disease progression in individual patients is the inclusion of age as a covariate (Section 6). Several major clinical indices used to predict mortality in sepsis (such as APACHE II score) include age as covariate. Chronologic age is a well-documented risk factor for death from sepsis that is independent of the severity of disease[13,64]. An active area of current immunologic research is the effect of age on the immune response. Additional studies will be necessary to include the effects of age on innate and cognate immune mechanisms in sepsis.

## Conclusion

Much work remains to be done to refine and validate the base theoretical model in robust animal studies of sepsis (such as rat cecal ligation and puncture) and in clinical studies. In particular, future studies will need to extend the current cross-sectional observations from relatively small numbers of healthy individuals to longitudinal studies of large numbers of acutely-ill sepsis patients (such as the PROWESS data set). Recent developments in our understanding the critical role of humoral and cellular innate immune mechanisms in sepsis outcomes need to be reflected in the model. Finally, our understanding of perturbations in basic physiologic mechanisms in sepsis is still maturing: For example, the concept of septic shock was recently expanded to include recognition of precursor states, such cryptic shock, through measurement of objective, clinical biomarkers, such as arterial lactate[10]. Another example has been the recent recognition of a continuum of endothelial activation, endothelial damage, increased vascular impedence and disseminated intravascular coagulation, possibly represented by the surrogate of plasma protein C levels[50,65-68]. Selective incorporation of clinically useful surrogates such as these into robust, dynamical models of sepsis holds the promise of enabling real-time, data-driven, treatment decisions[36,49,69]. Key clinical questions that such models might address include: Should this patient with CAP be admitted to hospital? Has this patient's clinical course deteriorated (or improved) in the last six hours? Would this patient benefit from activated protein C infusion? Would this patient benefit from early goal directed therapy?

## Methods

### Collection of clinical data

Physiologic values (Body mass (kg), height (cm), lung capacity (L), fasting glucose concentration (mmol/L), rate of blood circulation (by echocardiography, in L/min), and cardiac stroke volume (by echocardiography, L) were measured in 82 healthy volunteers at the Moscow State Medical Academy (Russia, courtesy of Dr. V. K. Korneenkov), and were collected in compliance with the Helsinki Declaration, and approval of the Institute of Numerical Mathematics at the Russian Academy of Sciences and Moscow State Medical Academy (Moscow, Russia).

Retrospective cardiovascular and cancer mortality information was obtained from public records 

## Competing interests

The author(s) declare that they have no competing interests.

## Authors' contributions

SMZ developed the mathematical model, identified the clinical data, and wrote the initial draft. SFK contributed the sepsis domain expertise and associated text. DG contributed the link to advances made in ecological metabolic scaling and associated text, and coordinated general edits and preparation of the final manuscript. All authors read and approved the final manuscript.
